# Expression of VPAC1 in a murine model of allergic asthma

**DOI:** 10.1186/1745-6673-8-28

**Published:** 2013-10-10

**Authors:** Hans D Lauenstein, David Quarcoo, Tobias Welte, Armin Braun, David A Groneberg

**Affiliations:** 1 Department of Pulmonary Medicine, Hannover Medical School, Hannover, Germany; 2 Fraunhofer Institute of Toxicology and Experimental Medicine, Hannover, Germany; 3 Institute of Occupational Medicine, Social Medicine and Environmental Medicine, Medical School, Goethe-University Frankfurt, Frankfurt, Germany

**Keywords:** VIP, VPAC1, Neurotransmitter, Asthma, Allergy

## Abstract

Vasoactive intestinal polypeptide (VIP) is a putative neurotransmitter of the inhibitory non-adrenergic non-cholinergic nervous system and influences the mammalian airway function in various ways. Hence known for bronchodilatory, immunomodulatory and mucus secretion modulating effects by interacting with the VIP receptors VPAC1 and VPAC2, it is discussed to be a promising target for pharmaceutical intervention in common diseases such as COPD and bronchial asthma. Here we examined the expression and transcriptional regulation of VPAC1 in the lungs of allergic mice using an ovalbumin (OVA) -induced model of allergic asthma. Mice were sensitized to OVA and challenged with an OVA aerosol. In parallel a control group was sham sensitized with saline. VPAC1 expression was examined using RT-PCR and real time-PCR studies were performed to quantify gene transcription. VPAC1 mRNA expression was detected in all samples of OVA-sensitized and challenged animals and control tissues. Further realtime analysis did not show significant differences at the transcriptional level.

Although the present studies did not indicate a major transcriptional regulation of VPAC1 in states of allergic airway inflammation, immunomodulatory effects of VPAC1 might still be present due to regulations at the translational level.

## Introduction

Respiratory diseases constitute a major part of occupational diseases. Next to asbestosis [1,2] and tuberculosis [3,4], bronchial asthma belongs to leading causes of the global burden of occupational lung disease [5-7] and further insights into the underlying mechanisms of the disease are needed. Among these mechanisms, pathways of the neuro-immune axis may play a key role. In this respect, vasoactive intestinal peptide (VIP) is a putative neurotransmitter of the non-adrenergic non-cholinergic nervous system in the respiratory tract [8]. VIP-immunoreactivity is present in cells of the tracheobronchial smooth muscle layer, in the walls of pulmonary and bronchial vessels, around submucosal glands, in the lamina propria and in pulmonary ganglia [8,9]. VIP immuno reactive nerve fibres are found as branching networks in the respiratory tract [8] and they are decreasing in frequency as the airways become smaller, but extend to peripheral bronchiols [10,11]. VIPergic nerves are colocalized with cholinergic nerves and VIP immunoreactivity is also present in sensory nerves [8,12].

VIP is a highly abundent and pleiotrophic mediator, known for various effects, including bronchodilatation, modulation of mucus secretion and immunomodulation. VIP is therefore a promising target for future therapy in chronic inflammatory lung diseases [13].

VIP binds predominantly to the two VIP-receptors VPAC1 and VPAC2. Antiinflammatory effects have been demonstrated to be mediated by the VPAC1 receptor *in vivo* and *in vitro*. VPAC1 activation was shown to inhibit the release of inflammatory mediators from macrophages like IL-6, TNF-alpha, IL-12 and NO and increase of IL-10 in a murine model of septic shock. In contrast to VPAC2, VPAC1 also inhibits the proliferation of activated T cells and the expression of costimulatory molecules like B7.1 and B7.2 [14]. A reduction of VPAC1 expression could be shown for T cells in case of activation [15].

Since VPAC1 was suggested to play the major role in the antiinflammatory action of VIP [16] and anti-inflammatory effects were recently described for the receptor PAC1, the present study assessed the expression and regulation of the receptor VPAC1 [17] in a murine model of asthma on the transcriptional level.

## Material and methods

### Asthma Model and preparation of lung tissue samples

**Figure 1:**
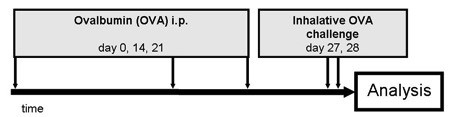
Treatment protocol for the murine asthma model; OVA sensitization via i.p administration of 10 mg OVA on days 0, 14 and 21 followed by aerosolic challenges with an 1% OVA aerosol for 20 min on days 27 and 28; sacrifice on day 29.

Female BALB/c mice were sensitized to ovalbumin (OVA) as previously described [17] by administration of 10 μg OVA and 1,5 mg Alum (Pierce, Rockford, USA) in 50 μL saline by intraperitoneal injection on days 0, 14 and 21 (n = 16) (Figure 1). The allergic response in the respiratory tract was induced by the exposition of mice to 1% OVA aerosol for 20 min on days 27 and 28. 24 hours after the last challenge the mice were sacrificed by an overdose of Narcoren, (Merial, Hallbergmoos, Germany). Lungs were then removed. The upper right lobe was dissected and snap frozen in liquid nitrogen. The samples were stored at −80°C until further analysis. The animal study was performed according to internationally recognized guidelines and approved by the local authorities (Hannover).

### Generation of cDNA

As previously described [17], the lung samples were homogenized using fastprep (Bio101, Carlsbad, USA) and total RNA was isolated using the RNeasy mini kit (Qiagen, Hilden, Germany) according to the customer’s manual. Quantity of RNA was controlled photometrically by OD 260/280 nm.

900 ng RNA were transcribed into cDNA using the omniscript RT-PCR kit (Qiagen, Hilden, Germany). RNA was denatured at 65°C for 5 minutes. After cooling to room temperature, buffer RT, random primers, RNase inhibitor, and omniscript for the RT-PCR were added. The RNA was transcribed at 37°C for 90 min and the reaction was stopped by heating up to 95°C for 5 min. Each RNA sample was used for two approaches: First for the RT-PCR and in a second approach as a RNA control for genomic DNA contamination in which samples were treated like the RT-PCR samples but without the transcriptase. Both, the cDNA and the RNA control were immediately used for the real time-PCR.

### Quantitative real time PCR

The relative amounts of cDNA were quantified using the Light Cycler Fast Start SYBR Green Kit (Roche, Mannheim, Germany) according to the customer’s protocol. Porphobilinogen deaminase (PBGD) was used as a house keeping gene. The DNA was amplified using the following conditions: 10 in 95°C; 45 cycles 1 s 95°C, 10 s 68°C, 9 s 72°C. The pureness of the products was verified via a melting curve. The products were visualized via gel electrophoresis.

All primers were produced by MWG (Ebersberg, Germany) and designed intron spanning to exclude the amplification of genomic DNA in case of contaminations. The PCR products were verified by DNA agarose gel and by sequencing by the company GATC biotech (Koblenz, Germany). The primer sequences are shown in Table 1.

**Table 1:** Primer sequences

**Gene**	**Primer forward**	**Primer reverse**	**Product size**
PBGD	cacgatcctgaaactctgct	agatggtccagaagatgaccc	224
PAC1	ttcactactgcgtggtgtccaact	atatcccagcatcccgcatcatca	228

## Results and discussion

**Figure 2:**
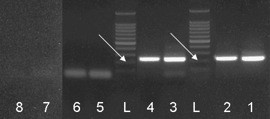
VPAC1 gel electrophoresis; L = 100 bp DNA ladder; arrows point at 200 bp band; slot 1 = OVA sensitized (OVs) PBGD; slot 2 = OVs VPAC1; slot 3 = sham sensitized (SHs) PBGD; slot 4 = SHs VPAC1; slot 5 = OVs PBGD RNA control (RC); slot 6 = OVs VPAC1 RC; slot 7 = SHs PBGD RC; slot 8 = SHs VPAC1 RC; expected products: PBGD = 224 bp; VPAC1 = 228 bp.

Specific PCR products for VPAC1 and PBGD were found in the lungs of both, the OVA sensitized and the saline sensitized control group. The products were identified to be specific by gel electrophoresis (Figure 2) and by melting curve analysis (data not shown).

**Figure 3:**
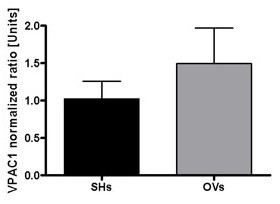
Normalized ratio of VPAC1 expression in lung tissue; VPAC1 fluorescence signal normalized to PBGD; OVs = OVA sensitized and challenged group, SHs = sham sensitized group.

To assess quantitative changes of VPAC1 mRNA expression, the VPAC1 fluorescence signal was normalized to the house keeping gene PBGD using the program rel quant (Roche) and displayed as relative unit. Though the real time PCR results tend to show a slight increase of VPAC1 expression in the lungs of OVA sensitized animals compared to the sham sensitized group (Figure 3), no significant difference can be detected using statistical approaches.

Although a clear alteration in the VPAC 1 expression on RNA level could not be detected in the present study, differential VPAC1 expression in terms of inflammatory mechanisms had been shown before. In this respect, in an *in vitro* model with different subsets of T cell subsets the VPAC1 expression was linked to an inhibition of proliferation and the expression of costimulatory molecules [14]. VPAC1 was also shown to be downregulated in case of activation of T cells [15].

VPAC1 expression was shown in various cells of the immune system like T cells and B cells in other compartments of the body such as the intestinal system [18]. Also the infiltration of these VPAC1-positive cells during an inflammation like in ulcerative colitis and crohn’s disease was shown to increase the amount of VPAC1 positive cells in the inflammed tissue [18].

According to literature it could be hypothesized, that VPAC1 might play a role in the pathogenesis of bronchial asthma, for effects mediated by VPAC1 are present in allergic diseases such as asthma or atopic dermatitis. Nevertheless we did not find a regulation of VPAC1 on the transcriptional level so far. Therefore, it is necessary to take a more detailed look into the lung tissue on the single cellular level and VPAC1 positive cells and the expression of VPAC1 should be quantified for each cell population separately. Overlaps of alterations in VPAC1 expression in specific cell populations by the influx of VPAC1 positive cells could thereby avoided. Also, regulation may take place at the level of translation and future studies should access this aspect.

Interestingly, the VPAC1 related receptor PAC1 was recently shown to be differentially expressed in a murine model of ovalbumin-induced asthma [17]. In this study an increased PAC1R mRNA expression was present in lung tissue under allergic conditions. In the state of an asthmatic reaction, PAC1R-deficient mice (PAC1R(−/−)) and to BALB/c mice treated with the specific PAC1R agonist maxadilan, PAC1R deficiency resulted in increased inflammatory effects, while agonistic stimulation of It was therefore concluded that anti-inflammatory effects can be achieved via PAC1 receptor and that PAC1receptor agonists may represent a promising target for an anti-inflammatory therapy.

In summary, the present analysed the expression and transcriptional regulation of VPAC1 in a murine model of allergic asthma using an established ovalbumin protocol. While VPAC1 mRNA expression was detected in all samples realtime analysis did not show significant differences at the transcriptional level. Therefore further studies should quantify VPAC1 expression on the level of single respiratory cells using modern tools of biochemistry [19], molecular biology [20], cell biology [21][22] and morphology [23].

## Competing interests

The authors declare that they have no competing interests.

## Authors’ contributions

HDL, DQ, TW, AB, DAG have made substantial contributions to the conception and design of the study, acquisition of the data and interpretation. HDL, DQ, TW, AB, DAG have been involved in drafting and revising the manuscript. All authors have read and approved the final manuscript.
